# Profiling of Peripheral TRBV and CD4+CD25+ Treg in CHB Patients with HBeAg SC during TDF Treatment

**DOI:** 10.1155/2023/1914036

**Published:** 2023-01-10

**Authors:** Jiezuan Yang, Haifeng Lu, Baikun Chen, Lili Jiang, Hua Zhang, Ping Ye, Linfeng Jin

**Affiliations:** ^1^The First Affiliated Hospital, Zhejiang University School of Medicine, Hangzhou 310003, China; ^2^State Key Laboratory for Diagnosis and Treatment of Infectious Diseases, Hangzhou 310003, China; ^3^National Clinical Research Center for Infectious Diseases, Hangzhou 310003, China; ^4^School of Laboratory Medicine and Life Science, Wenzhou Medical University, Wenzhou 325035, China; ^5^Department of Microbiology and Immunology, School of Basic Medicine, Wenzhou Medical University, Wenzhou 325035, China

## Abstract

**Background:**

It is lacking that markers could predict the prognosis of chronic hepatitis B (CHB) subjects during antiviral treatment, and the related cellular immune mechanism is not fully evaluated.

**Aim:**

To explore the comprehensive profile of T cell receptor *β*-chain (TRBV) and CD4+CD25+ regulatory T cell (Treg) in peripheral blood of CHB patients with HBeAg seroconverting (SC) during tenofovir disoproxil fumarate (TDF) treatment.

**Methods:**

The frequency of CD4+CD25high+ Treg and number of skewed TRBV in 20 HBeAg positive patients were determined at baseline and following every 12 weeks during 96-week TDF treatment. The relationship among serum alanine aminotransferase (ALT) level, HBV DNA load, Treg frequency, and the number of skewed TRBV, respectively, was analyzed for CHB patients. Receiver operative characteristic curve was applied to analyze their diagnostic value for HBeAg SC.

**Results:**

The number of skewed TRBV at week 48, Treg frequency at week 72, and ALT level at baseline could predict the HBeAg SC or non-SC in CHB patients during 96-week TDF treatment. Moreover, the positive correlation between ALT or HBV DNA and Treg levels or skewed TRBVs was significant in the SC group, but not in non-SC.

**Conclusions:**

The predictive cutoff value of ALT for HBeAg SC was 178 U/L at baseline. Moreover, the ALT, Treg, and TRBV families would be associated with the prognosis and pathogenesis of CHB patients during TDF treatment.

## 1. Introduction

Chronic persistent hepatitis B virus (HBV) infection could result in liver cirrhosis, hepatocellular carcinoma (HCC), and even liver failure [1, 2]. It is estimated that about 2 billion people around the world carrying HBV in their life, of which 250 million people (including 2 million Americans) would develop into chronic HBV infection [3]. Although the hepatitis B (HepB) vaccination has prevented individual from HBV infection, no therapeutic vaccine now is available for HepB, and nearly 800 thousand individuals die from HBV-related diseases every year. Especially, the HBV-related HCC is the third highest cancer-related mortality in the world [4].

At present, antiviral therapy is considered to be a main means to delay the progression of chronic HBV infection. As a first-line drug for the treatment of chronic HBV infection, the tenofovir disoproxil fumarate (TDF) could mediate antiviral, antiproliferative, and immunomodulatory effects; however, its efficacy is limited in less than half of subjects treated, and the underlying reason is still vague [5–7]. Therefore, the immunological mechanism of chronic hepatitis B (CHB) patients who responded or nonresponded to antiviral therapy needs further evaluated.

Hepatitis B e antigen (HBeAg) is a vital indicator of antiviral treatment and is related to HBV replication and infectivity, and it can enter the umbilical cord blood through the placenta and result in vertical transmission (from mother to baby) [8]. Moreover, as a tolerogen, HBeAg can weaken the immune response of newborns to HBV, inhibit the cytotoxic activity of host T lymphocytes, and lead to immune tolerance to HBV infection and chronicity of HBV infection [9, 10]. Therefore, for chronic HBV infection, HBeAg transformation usually is one of a valuable indicator to predict clinical prognosis of CHB patients during antiviral treatment [11, 12]. Moreover, the CHB patients obtaining HBeAg seroconverting (SC) are considered to be the satisfactory goal of anti-HBV treatment [13].

Host immune response to HBV plays a key role in the pathogenesis of chronic HBV infection; simultaneously, the immune response induced by antiviral therapy against HBV also is a vital role in viral clearance [14, 15]. CHB patients with HBeAg are receiving TDF treatment, which can induce dynamic changes in immune-related parameters [16]. However, the predictive value of these parameters for HBeAg SC in CHB patients is still needed to determine [17]. In order to explore the immune-related predictors of HBeAg SC, we profiled the dynamic changes of TRBV features and CD4+CD25+ Treg frequencies and the serum ALT levels in CHB patients during 96-week TDF treatment. In addition, we evaluated the correlation between serum ALT level or HBV DNA load and the number of skewed TRBV or Treg frequency.

## 2. Methods

### 2.1. Subjects

A total of 58 outpatients (subjects) with CHB were selected from the Department of Infectious Disease, the First Affiliated Hospital, Zhejiang University School of Medicine (FHZJU), between November 2014 and May 2019. The inclusion criteria of CHB subjects were according to the definitions from our previous report and the guideline of prevention and treatment for chronic hepatitis B (2010 version) [18], and more inclusion and exclusion criteria were also described in other's reports [19]. The baseline demographic and clinical characteristics of the subjects are shown in Table 1.

At the end of the study, due to various uncontrollable factors (during the 96-week period, 16 subjects could not be contacted, 10 subjects switched to other nucleotide analogues for the cost, and 12 subjects dropped out for unknown reasons), there were only 20 subjects completed 96 weeks of TDF treatment and with data required for the study. In detail, all subjects were orally treated with 300 mg of TDF-naive daily and were scheduled to continue the TDF treatment for 96 weeks. The twenty HBeAg (+) CHB subjects were divided into HBeAg seroconverting (SC, *n* = 9) or non-SC (*n* = 11) groups, depending on whether they had undergone HBeAg SC by week 72 of TDF treatment, that was HBeAg loss (quantitative HBeAg < 0.18 PEIU/mL) and positive for anti-HBe antibody (quantitative anti-HBe < 1.00 S/CO) [20]. This protocol was approved by the ethics committee of the FHZJU (Reference No. 2015-497), and all subjects gave informed consent. The study was also conducted according to the guidelines of the Declaration of Helsinki.

### 2.2. Sample Preparation

The enrolled subjects were collected 5 mL of fasting peripheral whole blood in the morning; the peripheral blood samples were collected at baseline and the follow-up visits at weeks 12, 24, 36, 48, 60, 72, 84, and 96. Additionally, fifteen healthy controls (HCs) were selected as controls and were sex- and age-matched with the CHB group. The recruited HCs had no previous history or current evidence of liver disease (they were negative for all HBV serological markers), and all known clinical laboratory indices are within the normal reference range, and some indicators are also shown in Table 1.

### 2.3. PBMC Isolation and cDNA Synthesis

Peripheral blood mononuclear cells (PBMCs) were isolated from 5 mL blood (treated with EDTAK2 anticoagulant) using Ficoll-Paque (STEMCELL Technologies, Vancouver, Canada) density gradient separation. Total RNA was extracted from PBMCs using the SV Total RNA Isolation System (Promega, Madison, WI, USA) according to the manufacturer's instructions. Subsequently, the total RNA was reverse transcribed into cDNA, and the details about total RNA extraction and cDNA synthesis were presented in our previous report (including RNA quality control and quantification) [21]. HBV genotypes were confirmed with sequence detection via PCR; the detail was shown in our previous report [22, 23].

### 2.4. Detection of Biochemical Indices and HepB Serological Markers

Subjects enrolled were followed up every 12 weeks for routine inspection and clinical laboratory testing. HBV serological markers were determined with enzyme immunoassays (Abbott, Chicago, IL, USA) according to the test guidance. Routine liver biochemistry, serum ALT, albumin (ALB), and total bilirubin (TBiL) were detected using an automatic biochemical analyzer (AEROSET, Abbott, Chicago, IL, USA). Serum HBV DNA load was quantified using Cobas Amplicor/Cobes TaqMan HBV test (Roche Diagnostics, Indianapolis, IN, USA) with a detection limit of 30 cps/mL according to the manufacturer's instructions. These indices were quantified in the clinical laboratory center of the FHZJU.

### 2.5. Flow Cytometric Analysis of Treg Frequencies

The percentage of CD4+CD25+ regulatory T cells (Treg) per CD4+ T cells was assayed using flow cytometry; in briefly, the CD4+CD25+ cell subset was gated from a mononuclear cell subset of peripheral blood, and the frequencies of CD4+CD25high+ (fluorescence intensity of CD25 > 50) cell subset in CD4+ T cell were evaluated as CD4+CD25+ Tregs, which was also described in our previous report [24].

### 2.6. Analysis and Identification of Skewed TRBV Families by GMSP

GoTaq qPCR Master Mix (Promega) was used for the gene melting spectral pattern (GMSP) assay. A 25 *μ*L master mix for each of the 28 reactions contained 0.4 mM primers and 50~150 ng of cDNA as a template to produce GMSPs as previously described [21]. The TRBV families were divided into three categories: oligoclonal, monoclonal, and normal expansion; more classification rules were described in our previous reports [18, 25]. In briefly, based on the profile of PCR-product melting curve analysis, (i) “oligoclonal” presented a main peak and other small peaks with a height less than 5/8 height of the main peak; (ii) “monoclonal” presented one main peak and, perhaps, with a short small peak with a height less than 3/8 height of the main peak, and (iii) if the height of any secondary peak was more than 5/8 height of the main peak, the TRBV family was considered to be “normal expansion.” The TRBV family with oligoclonal or monoclonal manifestation was classified into skewed family, and the number of skewed families was counted for each CHB subject [26].

### 2.7. Statistical Analysis

SPSS for all data (version 19.0, Chicago, IL, USA) and GraphPad Prism (version 8.0, San Diego, CA, USA) were used for statistical analysis and drawing. Normal distribution data were expressed as mean ± SD (standard deviation), and the no-normal data were described as mean ± SEM (standard error of the mean). The Mann–Whitney *U* test was used for nonpaired continuous variables with no-normal data. Spearman's bivariate correlation was used for correlation analysis of no-normal data. The categorical variables were analyzed by chi-square test or Fisher's test. The Kaplan-Meier method was used for survival (HBeAg SC) analysis, and log-rank test was used to compare the survival (HBeAg SC) differences among the 20 CHB subjects. *P* < 0.05 was considered statistically significant.

## 3. Results

### 3.1. Characteristics of Subjects

The subjects enrolled in this study had never received any anti-HBV therapy. No other liver-related complications detected during treatment, and no serious adverse events or hepatitis flares were observed during the total 96-week TDF treatment. HBeAg SC was observed beginning at week 12 and also occurred at follow-up treatment time points (one subject at week 12, two at week 24, three at week 36, six at week 48, seven at week 60, and nine at week 72). We found that the difference in HBV DNA levels between the SC and non-SC groups at baseline was significant (data not shown). During the process of TDF treatment, HBVDNA levels declined consistently by almost four log10 from baseline by week 12 in both the SC and non-SC groups and could not be detected after week 72 only in the patients with HBeAg SC.

### 3.2. Dynamic Change Number of Skewed TRBVs in SC and Non-SC Subjects

**Figure 1:**
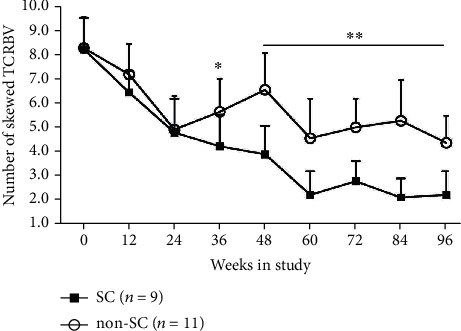
Dynamic change number of skewed TRBV families in CHB subjects with HBeAg seroconverting (SC) and non-SC during TDF treatment for 96 weeks. The number of skewed TRBV families was calculated by complying with the criteria listed in Methods. ^∗^*P* < 0.05 and ^∗∗^*P* < 0.01, the number of skewed TRBVs in HBeAg SC subjects compared to those in non-SC subjects at the same treatment time point. This significant difference began at week 36 and was analyzed using nonparametric tests.

Longitudinal study of the number of skewed TRBVs in HBeAg SC and non-SC subjects during TDF treatment for 96 weeks and the number of skewed TRBVs presented continuous declines in HBeAg SC and non-SC subjects. However, there was a significant difference in the number of skewed TRBVs between SC and non-SC subjects beginning at week 36 and following time points (Figure 1).

### 3.3. Similar Treg Frequencies between SC and Non-SC Subjects at Baseline

**Figure 2:**
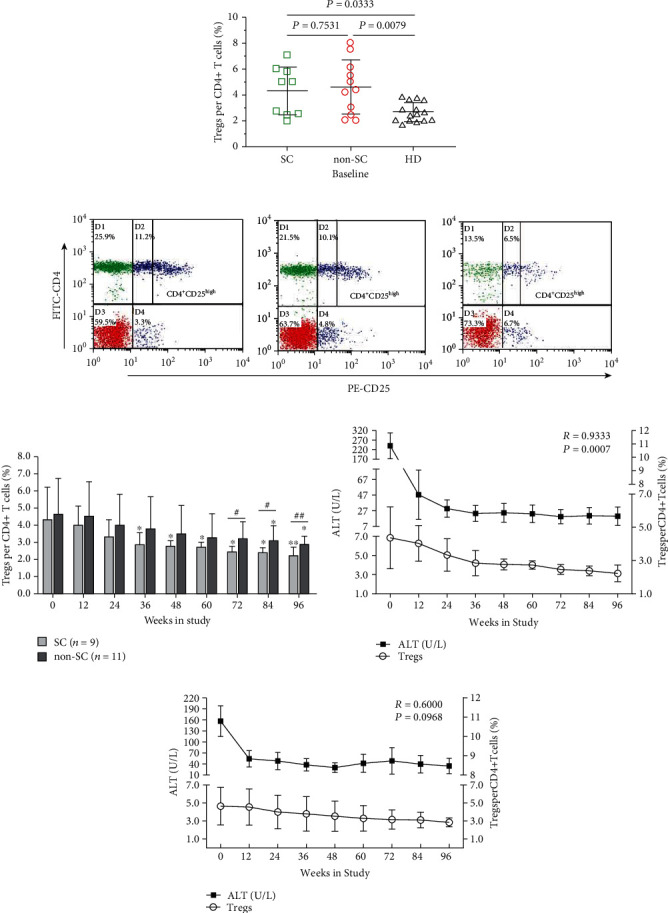
Association between the ALT levels and the Treg frequencies in HBeAg seroconverting (SC) and non-SC subjects during TDF treatment. (a) Peripheral CD4+CD25+ Treg frequencies in HBeAg SC, non-SC subjects, and healthy control (HC). Data are expressed as a scatter diagram in which the midpoint of the black solid line is the mean of Treg frequencies (Treg per CD4+ T cells, %), and the upper and lower horizontal lines represent the standard deviation (SD). The *P* values of multiple comparisons were calculated using the Kruskal–Wallis *H* nonparametric test. (b) Representative scatter plots were obtained by flow cytometry labeled antibodies against CD4 and CD25. The number of CD4+CD25high (CD4+CD25high+) Treg cells per CD4+ T cells was evaluated (fluorescence intensity of CD25 > 50). (c) Longitudinal changes of CD4+CD25+ Treg percentages in HBeAg SC and non-SC subjects during TDF treatment. ^∗^*P* < 0.05 and ^∗∗^*P* < 0.01 when comparing to those in SC or non-SC subjects at baseline, respectively. #*P* < 0.05 and ##*P* < 0.01 when comparing the Treg percentages between the HBeAg SC and non-SC subjects at the same treatment time point using nonparametric tests. Association is between the ALT levels and Treg percentages in HBeAg SC (d) and non-SC (e) subjects. The *x*-axis indicates the different treatment time points; (d) and (e) on the left *y*-axis show the ALT levels, and the right *y*-axis shows the Tregs per CD4+ T cells (%). Correlations were analyzed using a Spearman correlation analysis.

We quantified the CD4+CD25high+ (fluorescence intensity of CD25 > 50) Treg subset percentage per the total CD4+ population. There was no significant difference in the frequency of Tregs between HBeAg SC and non-SC subjects at pretreatment (baseline; mean, SC = 4.37%; non-SC = 4.65%, *P* = 0.7531); however, the frequencies in both groups were significantly higher than those of the healthy controls (HC, mean = 2.71%, *P* = 0.0333, 0.0079) (Figure 2(a)). Additionally, a representative flow plot demonstrating the Treg (CD4+CD25high+) frequencies in peripheral blood from HBeAg SC, non-SC subjects, and HC is shown in Figure 2(b).

### 3.4. Dynamic Frequency of Tregs in SC and Non-SC Subjects

Dynamic changes in the Treg frequency during TDF treatment are displayed in Figure 2(c). The panel presented the Treg frequency in HBeAg SC and non-SC subjects at each time point during TDF treatment for 96 weeks. Following TDF treatment, the Treg frequency of each treatment time point gradually declined and compared with that at baseline; a significant reduction in frequency was found beginning at week 36 in the HBeAg SC group. However, the non-SC group showed a modest decrease in Treg frequency during TDF treatment until the beginning of week 84, while their frequencies were significantly lower than those at baseline. Furthermore, beginning at week 72, the Treg frequency in the SC group was significantly lower than those in the non-SC group at each treatment time point.

### 3.5. Relationship between ALT Levels and Treg Frequencies

There was a significant positive correlation between longitudinal changes in serum ALT levels and the circulating CD4+CD25+ Treg frequency in HBeAg SC subjects during 96-week TDF treatment (*R* = 0.9333, *P* = 0.0007; Figure 2(d)), but this correlation was not significant in the non-SC subjects (*R* = 0.6000, *P* = 0.0968; Figure 2(e)). However, there was a significant positive correlation between declines in serum HBV DNA load and Treg frequency in SC subjects during TDF treatment (*R* = 1.000, *P* < 0.001; Supplementary material Figure S1A), and a similar relationship was found in non-SC subjects (*R* = 0.933, *P* < 0.001; Supplementary material Figure S1B).

### 3.6. Relationship between HBV DNA Load or ATL Levels and the Number of Skewed TRBVs

**Figure 3:**
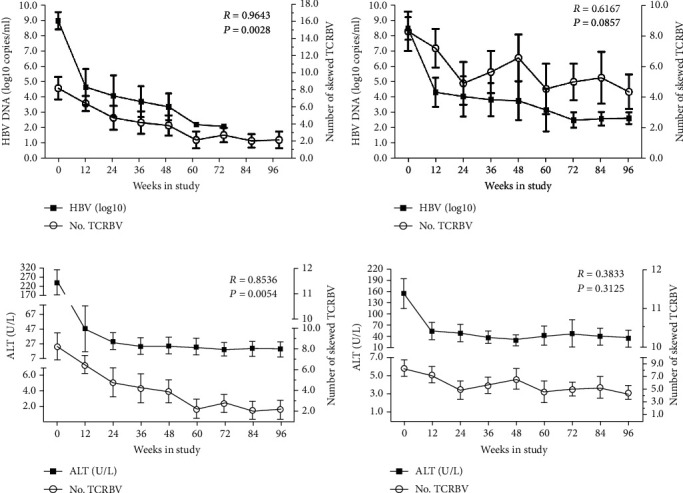
Association between the number of skewed TRBV families and the HBV DNA load or ALT level in HBeAg seroconverting (SC) or non-SC subjects during TDF treatment. The relationship between the HBV DNA level (log10 copies/mL) and the number of skewed TRBV families is shown for HBeAg SC (a) and non-SC subjects (b). The relationship between the ALT level (U/L) and the number of skewed TRBV families is shown for HBeAg SC (c) and non-SC subject (d). The *x*-axis indicates the different treatment time points; (a) and (b) on the left *y*-axis show the HBV DNA levels; (c) and (d) on the left *y*-axis show the ALT levels, and the right *y*-axis shows the number of skewed TRBV families. Correlations were analyzed using a Spearman correlation analysis.

The declining in the number of skewed TRBV families was associated with the decreases of serum HBV DNA load or ALT level. In the SC subjects, the number of skewed TRBV and HBV DNA load declined in parallel and was significant positive correlation (*R* = 0.9643, *P* = 0.0028; Figure 3(a)). In the non-SC, however, the correlation between them was not significant (*R* = 0.6167, *P* = 0.0857; Figure 3(b)). Similarly, there was a significant relationship between the number of skewed TRBV and the ATL level (*R* = 0.8536, *P* = 0.0054; Figure 3(c)) in the SC subjects, but not significant in the non-SC subjects (*R* = 0.3833, *P* = 0.3125; Figure 3(d)).

### 3.7. Dynamic ALT and HBeAg Levels in SC and Non-SC Subjects

**Figure 4:**
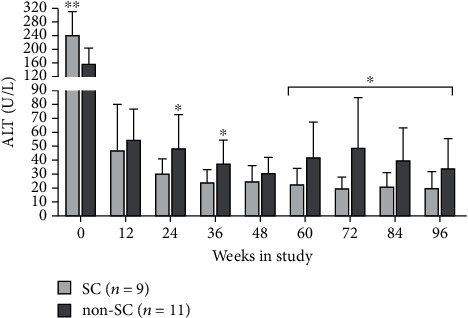
Dynamic change of ALT levels in CHB subjects with HBeAg seroconverting (SC) and non-SC during TDF treatment for 96 weeks. ^∗^*P* < 0.05 and ^∗∗^*P* < 0.01, the ALT levels in HBeAg SC subjects were compared to those in non-SC subjects at the same treatment time point. Statistical analysis uses nonparametric tests.

At baseline, the HBeAg SC subjects had a significantly higher level of ALT than that of non-SC subjects (*P* < 0.01). During the TDF treatment, the ALT level decreased consistently and reversed as significantly lower level of ALT than that of non-SC subjects at week 24 and keeping normal level (<48 U/L) in the following treatment period; however, in non-SC subjects, the ALT level rebound after week 60 (Figure 4). The HBeAg levels in SC subjects decreased consistently and could not be detected in all subjects after week 60, in contrast to these, the non-SC subjects whose HBeAg levels presented limited decline and were continuously detected all the time (Supplementary material Figure S2). Additionally, there was no significant difference in HBeAg levels between SC and non-SC subjects at baseline (Supplementary material Figure S3).

### 3.8. Effects of the Number of Skewed TRBV, Treg Frequency, and ALT Level on the HBeAg SC of CHB Subjects

**Figure 5:**
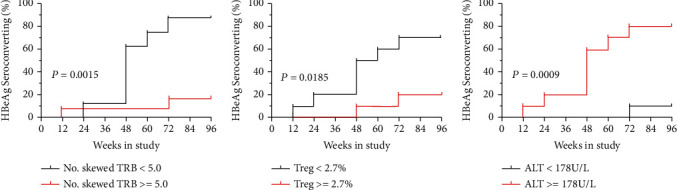
Prognostic value of the number of skewed TRBV families, Treg frequencies, and ALT levels in CHB subjects with or without HBeAg seroconverting (SC). The prognostic cutoff value of the number of skewed TRBV families at 48 weeks (a), Treg frequencies at 72 weeks (b), and ALT levels at baseline (c) in predicting the HBeAg SC of CHB subjects during TDF treatment for 96 weeks. The log-rank test was used to compare the difference of HBeAg SC among the CHB subjects during TDF treatment.

The Kaplan-Meier analysis showed that the 96-week HBeAg SC rate of CHB subjects with low number of skewed TRBVs (at week 48) was significantly higher than that of CHB subjects with high number of skewed TRBVs (HR = 7.367, 95% CI = 1.820~ 29.82, *P* = 0.0015; Figure 5(a)). Moreover, the 96-week HBeAg SC rate of CHB patients with low Treg frequency (at week 72) was significantly higher than that of the patients with high Treg frequency (HR = 4.959, 95% CI = 1.316~ 18.68, *P* = 0.0185; Figure 5(b)). Furthermore, higher ALT levels (baseline) are a favorable predictor of HBeAg SC in CHB subjects during TDF treatment (HR = 12.58, 95% CI = 3.293~ 48.05, *P* = 0.0009; Figure 5(c)). Additionally, the ROC of the number of skewed TRBV, Treg frequency, and ALT level were shown (Supplementary material Figure S4).

## 4. Discussion

Many studies have suggested that the immune response plays a vital role in controlling virus replication and persistence in the subjects with HBV infection [2, 27]. In the present study, we profiled the dynamic alteration of serum ALT level and CD4+CD25+ Treg frequency in CHB patients during 96-week TDF treatment and suggested that their characteristic profiles be associated with HBeAg SC of CHB subjects. Moreover, the number of skewed TRBV, Treg frequency, and serum ALT level could be used for differential diagnosis of HBeAg SC from non-SC patients with TDF treatment.

Tregs play a key role in the maintenance of immune tolerance and regulation of immune response [28, 29]. Treg frequency typically increases in CHB patients and is restored to normal levels in recovered CHB patients following effective antiviral treatment [2, 30]. The results in this study presented a significant positive correlation between serum ALT level and Treg frequency only in SC subjects during TDF treatment, which further confirms that a reduction in Treg frequency is associated with the process of HBeAg SC, and the subjects who did not achieve SC may be associated without being restored to normal Treg frequency [31, 32].

The negative immunomodulatory function of Tregs in HBV infection has attracted more and more attention. Treg under physiological conditions could regulate the immune balance and void excessive immune response in the host, which also is conducive to virus infection persistence [28]. The upregulated Tregs of CHB inhibit the activity and proliferation of HBV-specific CD4+ and CD8+ T cells and inhibit the secretion of IL-2 and IFN-gamma, so as to achieve immunosuppression and reduce host ability to clear the increasing HBV load [33]. Alternatively, the high HBV DNA load in CHB subjects was gradually declining when receiving effective antiviral treatment [26]. The high number of skewed TRBV indicates the imbalance of cellular immune status in host, which was not beneficial for the elimination of HBV [21]. The decreasing number of skewed TRBV indicates the host immune function gradually restored and experienced HBeAg SC in subjects during effective antiviral treatment.

In the course of antiviral treatment, CD4+CD25+ Treg frequency was declining in peripheral blood of CHB, and the decreasing degree of Tregs was greater in the HBeAg SC compared with that in the non-SC group, and the Treg frequency was higher in CHB subjects with active HBV replication [2, 34]. Furthermore, the Treg frequency is positively correlated with serum ALT level [30, 35], which was reconfirmed in our current study. The ALT levels usually reflect self-renewal metabolism of hepatocyte, and elevated serum ALT levels suggest the presence of active inflammation and the degree of hepatocyte injury caused by immune response against HBV [36, 37]. In the present study, the HBeAg SC subjects following antiviral treatment could develop normal serum ALT levels, and the decreasing levels of ALT were significantly positive correlated with the Treg frequency or the number of skewed TRBV, but no significance in the non-SC groups. The results suggested that the changing ALT level be associated with the cellular immune respond in patients with effective antiviral treatment and the damaged immunity gradually restored to new immune balance [38].

Additionally, this study further explored the diagnostic value of serum ALT level, Treg frequency, and the number of skewed TRBV for forecasting HBeAg SC of CHB patients during 96-week TDF treatment. Especially, the results showed that the cutoff value of ALT is 178 U/L (baseline) to predict HBeAg SC, with the sensitivity and specificity are 81.82% and 88.89%, respectively, which may be the first report on the usage of baseline ALT to predict the prognosis of CHB subjects during antiviral treatment. However, the sample size is small, and several other factors (viral, host, and even external) may influence the results; additionally, we will expand the sample size in a following study.

In summary, we present the dynamic profile of peripheral TRBV and Treg frequency for CHB subjects with or without HBeAg SC. The positive relationship between ALT level and Treg frequency was significant for SC subjects, but not for non-SC. Similarly, the positive correlation between HBV DNA load/ALT level and the number of skewed TRBV was also significant for SC subjects, but not for non-SC. Furthermore, the serum ALT level would be a potential target for distinguishing HBeAg SC from non-SC subjects with the ALT cutoff value (178 U/L) at baseline.

## Figures and Tables

**Table 1:** Clinical and virological parameters in enrolled subjects at baseline.

Variables	SC (*n* = 9)	Non-SC (*n* = 11)	HCs (*n* = 15)
Age (years)	29.78 ± 8.91	34.36 ± 9.11	32.60 ± 8.04
Gender (M/F)	7/2	9/2	13/2
ALB (g/L)	44.33 ± 1.80	45.27 ± 2.10	43.37 ± 3.88
ALT (U/L)	240.22 ± 67.84^∗∗^	156.71 ± 41.90	25.13 ± 5.40
AST (U/L)	146.33 ± 37.46	142.36 ± 33.51	21.87 ± 7.43
TBiL (*μ*mol/L)	19.67 ± 7.89	13.82 ± 3.67	10.40 ± 3.40
HBV genotype	4B/5C	5B/6C	ND
HBV DNA (lg, cps/mL)	8.91 ± 0.56	8.46 ± 0.73	ND
HBsAg (lg, IU/mL)	4.15 ± 0.53	3.86 ± 0.91	ND
HBeAg (lg, PEIU/mL)	2.67 ± 0.48	2.79 ± 0.36	ND

These values are expressed as mean ± SD, unless otherwise indicated. Normal values: ALT ≤ 48 U/L; AST ≤ 40 U/L; TBiL ≤ 26 *μ*mol/L; ALB 32~ 50 g/L. ALT: alanine transaminase; AST: aspartate aminotransferase; ALB: albumin; DNA: deoxyribonucleic acid; HBV: hepatitis B virus; HCs: healthy controls; TBiL: total bilirubin; ND: undetectable; non-SC: non HBeAg seroconverting; SC: HBeAg seroconverting. ^∗∗^*P* = 0.0033 vs. non-SC.

## Data Availability

All data are available from the authors upon reasonable request.

## Supplementary Materials

Supplementary Materials Figure S1: association between the HBV DNA loads and CD4+CD25+ Treg frequency in seroconverting (SC) and non-SC subjects. Associations between the HBV DNA loads and CD4+CD25+ Treg frequency in SC (A) and non-SC (B) subjects during TDF treatment. The *x*-axis indicates the different treatment time points; (A) and (B) on the *y*-axis show the HBV DNA loads, and the right portion of the *y*-axis shows the Tregs per CD4+ T cells (%). Correlations were analyzed using a Spearman correlation analysis. Figure S2: dynamic change levels of HBeAg in HBeAg seroconverting (SC) and non-SC subjects during TDF treatment for 96 weeks. In the below table, the amount in lower two lines presents the number of subjects entering the study at each time point during the TDF treatment for 96 weeks (study week shown at the first line in the below table). ^∗^*P* < 0.05 for the HBeAg of SC subjects compared to that in non-SC subjects. ND: not detected. Figure S3: serum HBeAg levels in seroconverting (SC) and non-SC subjects before antiviral treatment (baseline). Data are expressed as a scatter diagram in which the midpoint of the black solid line is the mean of HBeAg levels. Figure S4: HBeAg seroconverting (SC) discriminating receiver operating characteristic curve using the number of skewed TRB (A), peripheral Treg frequency (B), and serum ALT level (C).
