# Supplementary material for: Profiling of Peripheral TRBV and CD4+CD25+ Treg in CHB Patients with HBeAg SC during TDF Treatment
Source: J Immunol Res. 2023 Jan 10;2023:1914036. doi: 10.1155/2023/1914036 (PMC9845053; doi:10.1155/2023/1914036)
Supplement: Supplementary Materials — Figure S1: association between the HBV DNA loads and CD4+CD25+ Treg frequency in seroconverting (SC) and non-SC subjects. Associations between the HBV DNA loads and CD4+CD25+ Treg frequency in SC (A) and non-SC (B) subjects during TDF treatment. The x-axis indicates the different treatment time points; (A) and (B) on the y-axis show the HBV DNA loads, and the right portion of the y-axis shows the Tregs per CD4+ T cells (%). Correlations were analyzed using a Spearman correlation analysis. Figure S2: dynamic change levels of HBeAg in HBeAg seroconverting (SC) and non-SC subjects during TDF treatment for 96 weeks. In the below table, the amount in lower two lines presents the number of subjects entering the study at each time point during the TDF treatment for 96 weeks (study week shown at the first line in the below table). ∗P < 0.05 for the HBeAg of SC subjects compared to that in non-SC subjects. ND: not detected. Figure S3: serum HBeAg levels in seroconverting (SC) and non-SC subjects before antiviral treatment (baseline). Data are expressed as a scatter diagram in which the midpoint of the black solid line is the mean of HBeAg levels. Figure S4: HBeAg seroconverting (SC) discriminating receiver operating characteristic curve using the number of skewed TRB (A), peripheral Treg frequency (B), and serum ALT level (C). [file 1914036.f1.docx]

Supplementary materials

## Figure S1


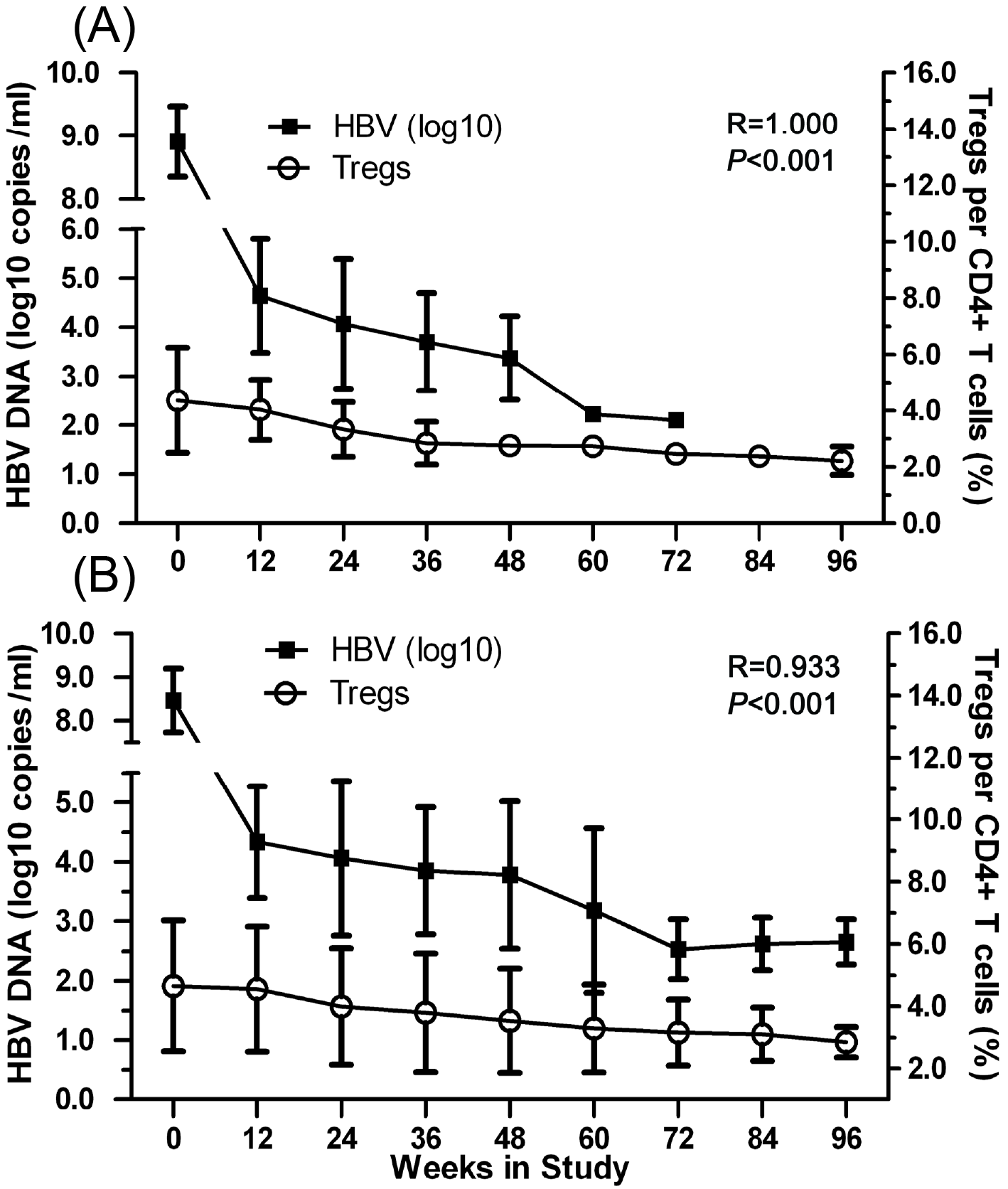


Association between the HBV DNA loads and CD4+CD25+ Treg frequency in seroconverting (SC) and non- SC subjects

Associations between the HBV DNA loads and CD4+CD25+ Treg frequency in SC (A) and non-SC (B) subjects during TDF treatment. The x-axis indicates the different treatment time points; A and B on the y-axis show the HBV DNA loads; and the right portion of the y-axis shows the Tregs per CD4+ T cellls (%). Correlations were analyzed using a Spearman correlation analysis.

## Figure S2


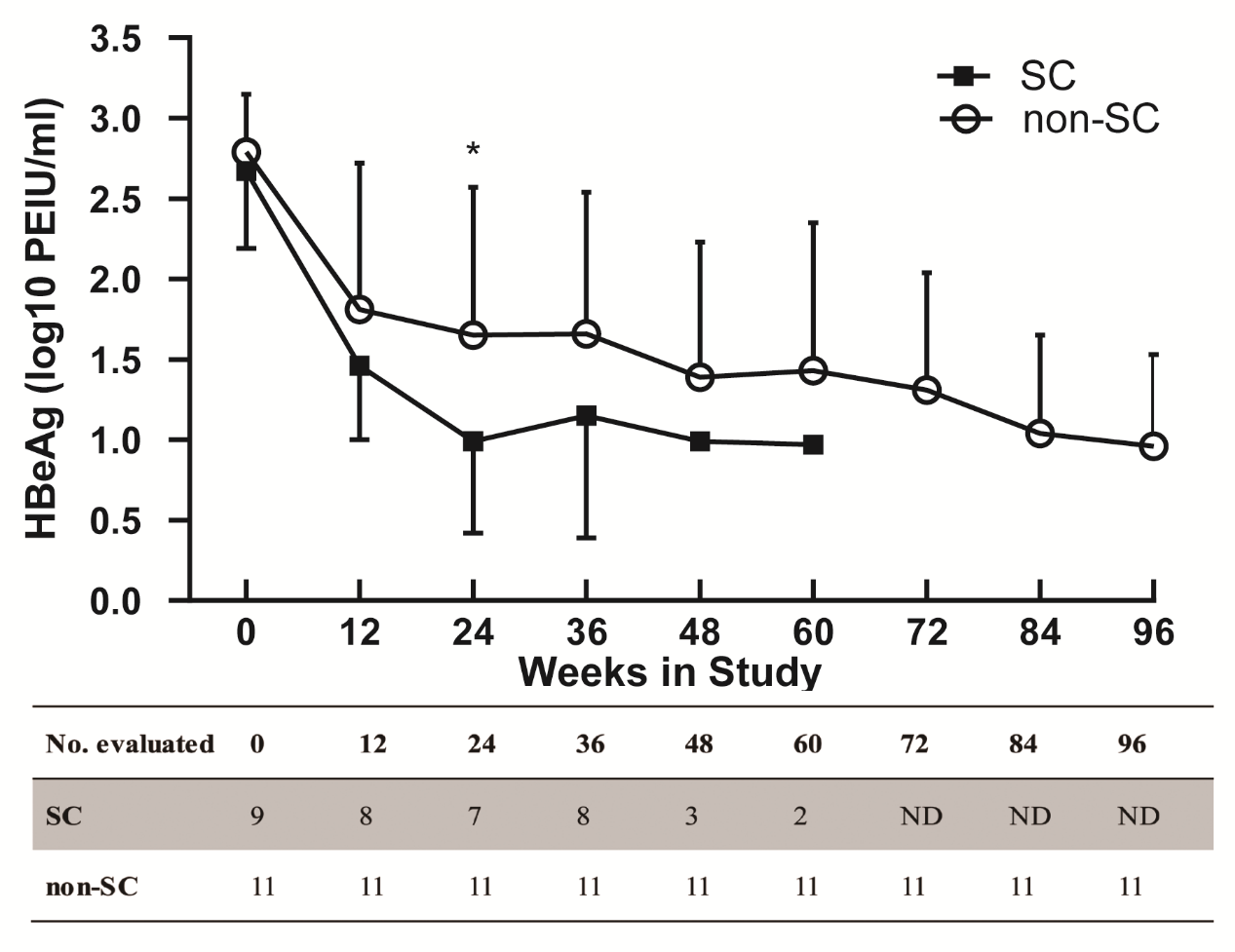


Dynamic change levels of HBeAg in HBeAg seroconverting (SC) and non-SC subjects during TDF treatment for 96 weeks

In the below table, the amount in lower two lines present the number of subjects entering the study at each time point during the TDF treatment for 96 weeks (study week shown at the first line in the below table).

*P<0.05 for the HBeAg of SC subjects compared to that in non-SC subjects. ND, no detected

## Figure S3


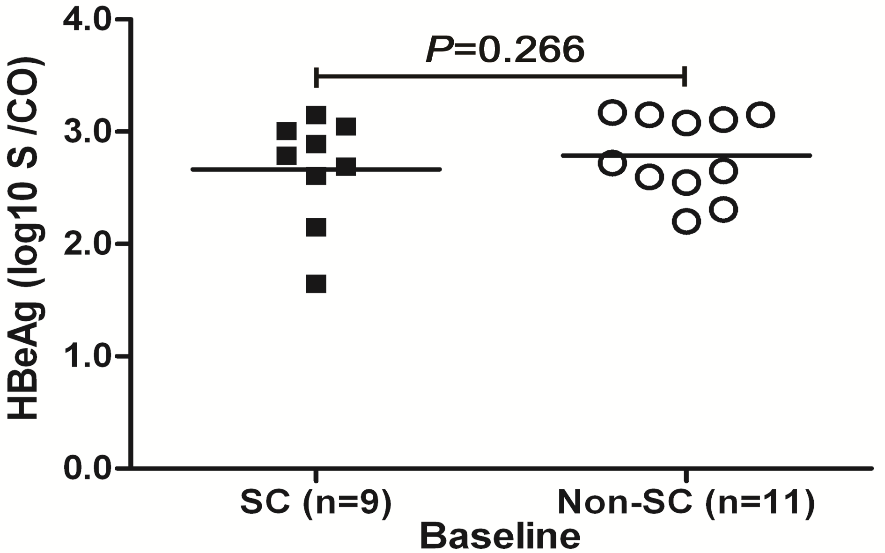


Serum HBeAg levels in seroconverting (SC) and non- SC subjects before antiviral treatment (baseline)

Data are expressed as a scatter diagram in which the midpoint of the black solid line is the mean of HBeAg levels.

## Figure S4


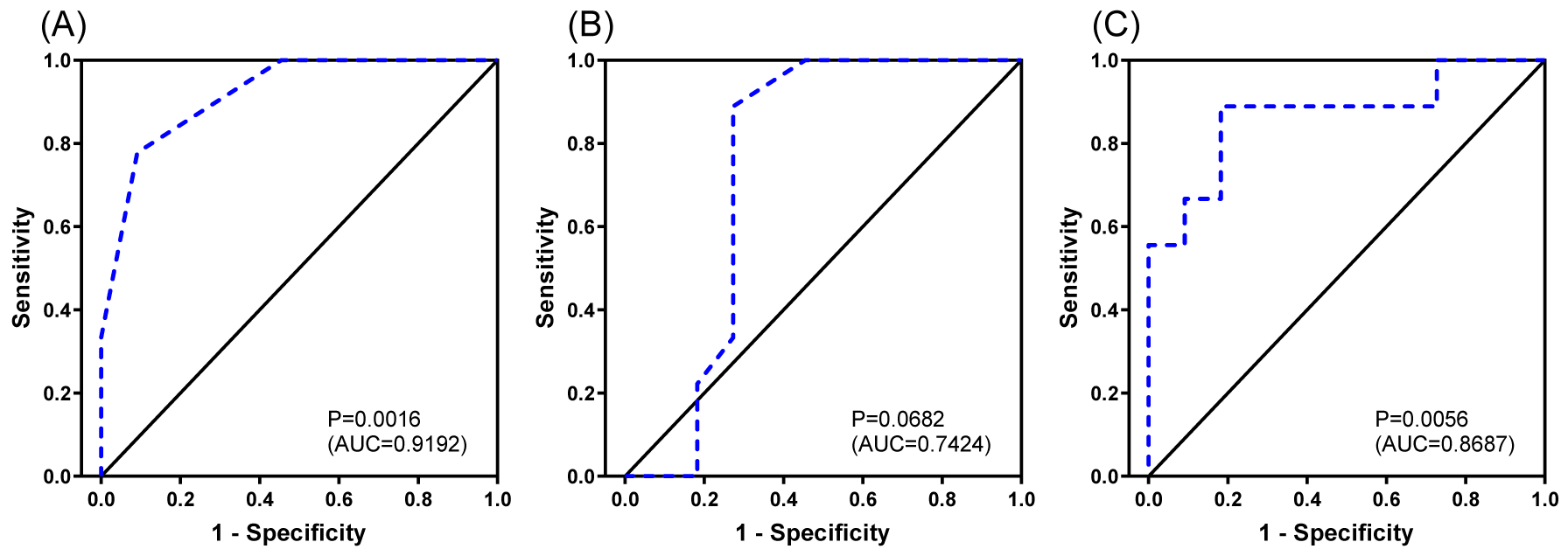


HBeAg seroconverting (SC) discriminating receiver operating characteristic curve using the number of skewed TRB (A), peripheral Treg frequency (B), and serum ALT level (C)
